# Consequences of Ischemic Preconditioning of Kidney: Comparing between Male and Female Rats

**Published:** 2012

**Authors:** Seyyed Meisam Ebrahimi, Nahid Aboutaleb, Maliheh Nobakht

**Affiliations:** 1 *Abhar School of Nursing, Zanjan University of Medical Sciences, Abhar, Zanjan, Iran *; 2 *Physioloy Research Centre and Physiology Department, Faculty of Medicine, Tehran University of Medical Sciences, Tehran, Iran*; 3 *Anatomy and Neuroscience Department, School of Medicine, Tehran University of Medical Sciences, Tehran, Iran *

**Keywords:** Ischemic preconditioning, Kidney, Reperfusion injury, Sex difference

## Abstract

**Objective(s):**

Ischemia-reperfusion injury (IRI) is a leading cause of kidney transplantation failure, and ischemic-preconditioning (IPC) is a protective method against the IRI. In the present study, the defensive effect of IPC on rats’ kidney was investigated and more importantly the differences between two genders were appraised.

**Materials and Methods:**

Thirty two Wistar rats were randomly allocated to four groups: group A (8 male IR), B (8 female IR), C (8 male IPC) and D (8 female IPC). Ischemia was induced by clamping of left renal arteries for 45 min in groups A and B. Rats in groups C and D experienced four cycles of 4 min arterial clamping followed by 11 min of de-clamping prior to the final 45 min of ischemia. 24 hr later, serum was provided to assess the blood urea nitrogen (BUN) and creatinine values. Also, renal tissues were obtained for histological measurements.

**Results:**

Induction of IPC in both male and female rats led to significant decrease in creatinine levels in comparison with sham groups (*P<*0.01). The same results were seen in BUN levels (*P<*0.01). However, there were no significant difference between two genders. Besides, histological protective effects of IPC was proved especially in female rats (*P<*0.01).

**Conclusion:**

Findings of our study confirmed that renal IPC reduces the damages in both genders especially females. Thus, the IPC procedure seems to be a useful method mainly in females.

## Introduction

Acute renal failure (ARF) induced by ischemia-reperfusion injuries (IRI) during kidney transplantation is an underlying problem that have led to drastic raise in the number of rejection cases (1-3). Several solutions have been proposed to decrease and control this harmful phenomenon (4). The manipulation of reperfusion process in donor kidney seems to be the most interesting and applicable approach. This method is called ischemic preconditioning or IPC. In this technique, numerous brief periods of ischemia-reperfusion are induced to the donor kidney prior to long-term ischemia, in order to prepare it for subsequent compulsive long-term ischemia during transplantation surgery. Various studies confirmed this method and implied that IPC critically decreased the long-term ischemia and also ischemia-reperfusion injuries in other body organs (4, 5).

However, researches showed that sex differences have intensive role in the severity and prognosis of renal diseases and IRI and also diseases progression are not the same between males and females in many cases (6). This issue could considerably influence the success of IPC in kidney and also the achievements of transplantation procedure.

However, there is no evidence about sex differences in the IPC of kidney. If there were any differences between genders, it would be seriously necessary to select the kidney donors based on their gender type and therefore, the success of kidney transplantation and survival of recipient patients would be more achievable.

Therefore, considering above information, we investigated and compared the gender differences in renal ischemic preconditioning.

## Materials and Methods

***Animals***

Experiment was performed on sexually mature male and female Wistar rats weighing 150-200 g. The animals were kept at the animal house of Tehran University of Medical Sciences (TUMS) in the condition of 12 hr lightness and 12 hr darkness and free access to standard rat chow and tap water. All experiments were approved by the Governmental Committee on Animal Welfare.

***General surgical preparation***

At first, rats were anesthetized with ketamine (60 mg/kg) and xylazine (10 mg/kg) IP. Throughout the anesthesia, the body temperature was monitored by a rectal probe and was maintained at 37°C using a heating pad. Then a midline laparotomy and right nephrectomy was performed. After 20 days, animals were divided to two distinct groups of males and females and each group was divided to two subgroups as follows (8 in each group): 1- ischemia reperfusion (IR) group: 45 min of left renal ischemia followed by reperfusion; 2- ischemic preconditioning (IPC) group: four cycles of 4 min of left renal ischemia separated by 11 min of reperfusion periods before the final 45 min of left renal ischemia followed by reperfusion.

Twenty-four hr later, animals were killed and renal tissues were extracted for histological assessments. Blood samples were collected from abdominal aorta and serum urea and creatinine levels were measured spectrophotometerically using a 704 Hitachi analyzer at 520 nm for BUN and 505 nm for creatinine. 

***Histological examination***

At the end of isolated perfusion, kidney samples were taken for histological evaluations. Kidney slices were fixed in 10% phosphate-buffered formalin overnight. After automated dehydration through a graded alcohol series, the slices were embedded in paraffin, sectioned at 4 µm and stained with hematoxylin-eosin. Blind analysis of morphological assessment was performed by an expert pathologist who was unaware of the experimental conditions and treatment that the animal had received.

The histological parameters evaluated were as tubular necrosis, tubular dilatation, interstitial edema, intracellular edema, and tubular cell brush integrity and cell detachment. A minimum of 8-10 fields for each kidney slide were examined and assigned for severity of changes with original magnification ×20. Morphological studies were conducted using an Olympus Photomicroscope (PROVIS AX70, Japan) equipped with a digital camera (DP11, Japan). Histological lesions were graded based on a semi-quantitative scale: 0- no abnormality, 1- mild lesions, affecting 10% of kidney samples, 2- moderate lesions, affecting 25% of samples, 3- severe lesions, affecting 50% of samples, 4- extreme lesions, affecting more than 70% of samples (7).

***Statistical analysisthis***

Data were analyzed using two-way ANOVA. The differences between groups were considered significant at *P<*0.01. All results were presented as mean±SD.

## Results

**Figure 1:**
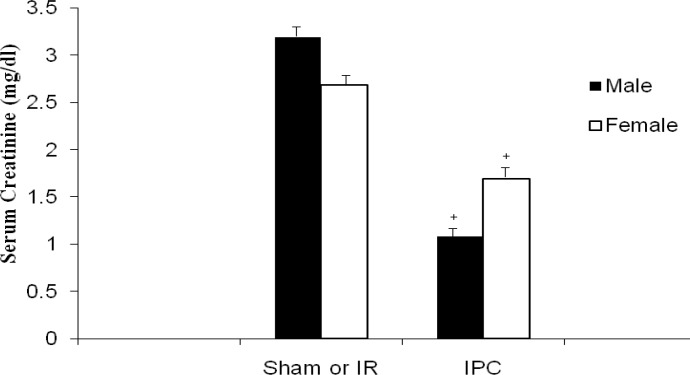
Creatinine levels in male and female ischemia reperfusion (IR) groups and ischemic preconditioning (IPC) groups. The values are represented as a mean±SD for 8 rats per group. ^+^Significantly different from co-gender IR group (*P<*0.01)

In male rats, serum creatinine levels decreased significantly in IPC group in comparison with IR group (µ±SD: 1.08 0.1 vs. 3.2 0.7 mg/dl, *P<*0.01). In female rats, the same results were seen (F-IPC vs. F-IR: µ±SD: 1.7 0.5 vs. 2.7 0.6 mg/dl, *P<*0.01). Creatinine level differences were not statistically significant between males and females in the similar groups (male-IPC vs. female-IPC e.g.) (Figure 1).

**Figure 2:**
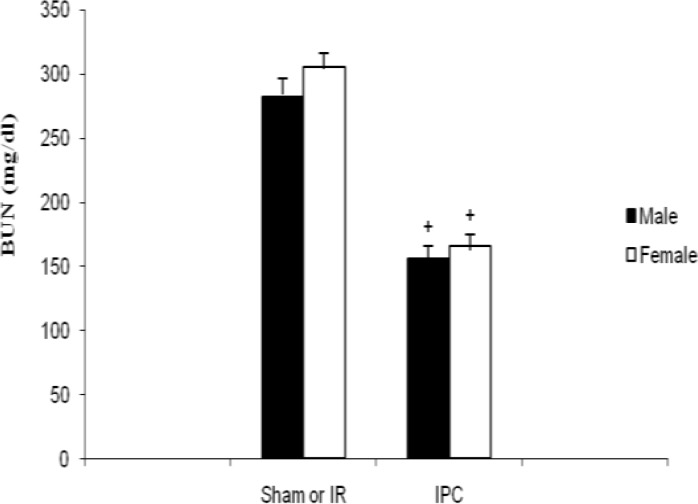
BUN levels in male and female ischemia reperfusion (IR) groups and ischemic preconditioning (IPC) groups. The values are represented as a mean±SD for 8 rats per group. ^+^Significantly different from the co-gender IR group (*P<*0.01)

Serum BUN level in male rats showed significant decrease in IPC in comparison with IR group (µ±SD: 155.7 31.9 vs. 282.14 34.3 mg/dl, *P<*0.01). In females, also the same results were seen (F-IPC vs. F-IR: µ±SD: 166 56.5 vs. 305.3 65.85 mg/dl, *P<*0.01). BUN level showed no significant differences between two genders in similar groups (Figure 2).

Results of histological examinations in both genders showed that the induction of IPC leads to improvement in the condition of renal tubules and glomerulus when compared with IR. In IPC males, demonstrations illustrated moderate tubular dilatation, focal glomerular necrosis, extent free space in corpuscle, loss of nuclei, predominates over morphological features of apoptosis (chromatin condensation and cell shrinkage), swelling tubular, less lumina congestion, moderate diffuse interstitial edema and dilatation of the tubular structure. Also, in IR males demonstrations illustrated the severity of all histological criteria. In IPC females, demonstrations showed mild tubular dilatation, focal glomerular necrosis, and extent free space in corpuscle, loss of nuclei, predominates over morphological features of apoptosis (chromatin condensatation and cell shrinkage), swelling tubular, lumina congestion, severe diffuse interstitial edema and dilatation of the tubular structure. But in IR females, severe tubular dilatation on one hand and on the other hand, moderate diffuse interstitial edema and moderate dilatation of the tubular structure was observed (Table 1). 

**Figure 3:**
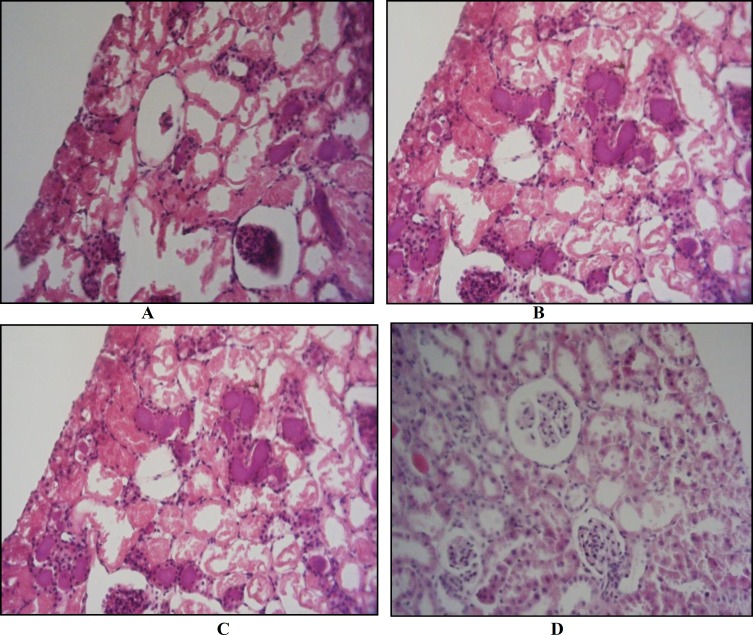
Renal histological microphotographs. Male kidney sections (A,C) and female kidney sections (B,D) were taken from ischemic-reperfusion (IR) rats groups (A, B) or ischemic-preconditioned (IP) group (C, D). In male ischemic-reperfusion group (A), there was severe tubular dilatation (▬), focal glomerular necrosis (●), much extent free space in corpuscle (■), loss of nuclei, chromatin condensation and cell shrinkage. In female ischemic-reperfusion group (B), there was severe tubular dilatation (▬), moderate focal glomerular necrosis (●), and moderate extent free space in corpuscle, moderate loss of nuclei, mild chromatin condensation and mild cell shrinkage. In male ischemic-preconditioned group (C), there was moderate tubular dilatation (▬) with loss of nuclei (■) in most of tubule, without swelling luminal congestion (e.g., moderate diffuse interstitial edema and moderate dilatation the tubular structure), less focal glomerular necrosis (●) and extent free space in corpuscle and predominates over morphological features of apoptosis (e.g., severe chromatin condensation and cell shrinkage). In female Ischemic-preconditioned group (D), there was mild tubular dilatation (▬) with loss of nuclei (■) in some of tubule, with swelling, luminal congestion (e.g., mild diffuse interstitial edema and mild dilatation the tubular structure), focal glomerular fragmentation (♦) and low extent free space in corpuscle and predominates over morphological features of apoptosis (e.g., moderate chromatin condensation and mild cell shrinkage)

Induction of IR in females caused fewer injuries to the kidney in comparison with males. Also, the induction of IPC in females led to more obvious improvement when compared with males (Figure 3).

**Table 1:** Histopathological evaluation of kidney damages in different groups based on scored criteria

Location	Criteria	M/IPC	F/IPC	M/IR	F/IR
Tubule	Dilatation	2	1	3	3
	Luminal digestion	2	0	3	2
	Nucleus changes	1	1	3	2
Glomeruli	Focal glomerular fragmentation	2	2	3	2
	Space in corpuscle	3	2	3	2
General	Swelling	1	2	3	2
Apoptosis	Cell shrinkage	2	1	3	1
	Chromatin condensation	2	2	3	1

Scoring: 0, none; 1, mild; 2, moderate; 3, severe; 4, extreme; M/IPC, male IPC group; F/IPC, female IPC group; M/IR, male IR group; F/IR, female IR group

## Discussion

The major new finding of this study was that an IPC protocol improved post-ischemic structural recovery in kidney in both female and male rats, but this recovery was more significant in females. Ischemic preconditioning is a powerful protective mechanism against ischemic injury in a variety of organs including the heart, brain, spinal cord, retina, liver, lung and muscolo-skeletal systems. Also, this phenomenon has protective effects against IR injuries (7-13). The difference of IPC between male and females has not been assessed very much. Pitcher *et al* investigated the difference in the myocardium tissue and showed that IPC has more protective effects in females (9). Also ُong *et al* stated that induction of IPC in Langendorff-perfused heart has more protective effects in female mice and implied that these effects disappeared after gonadectomy (10). Sex difference about the IR injuries has been studied in various organs and in most cases like brain, heart and spleen; the females were more resistant (11-13). In contrast, Gasbarrini *et al* concluded that females’ liver was more vulnerable to IRI which resulted in poor outcome of liver transplantation with female donors (14). Muller *et al *revealed that IR injuries of kidney are less in female rats and also showed that the injuries decreased after in-fertilizing the male rats and so androgen reduction (7). Two review studies (6, 15) revealed that the rate of renal disease progression is faster in males. Also, researches showed worse outcomes in chronic renal disease in males. This might be due to differences in the kidney structure, glomelular hemodynamic responses to stress and the direct cellular effects of sex hormones. Interestingly, selective estrogen receptor modulators (SERMs) like raloxifen have shown some reno-protective effects in animals and human (7, 8). This confirms the beneficial effects of estrogen on kidney. Kher *et al* suggested that females are protected against renal IR injury (16). Our study also confirmed these findings.

In this study, we compared the impact of IR on kidney between male and female rats and results corresponded to other studies like Kher *et al* (16). We also compared the effect of IPC on kidney and found out that IPC is effective in both genders. Previous studies assessed only male rats and did not investigate the females (17), however, in this study; both male and female rats were investigated and compared with one another. Our results showed that there are significant differences between IR and IPC groups regarding BUN and creatinine which means that IPC is effective in both genders. Also during histological examinations we found out that ischemia-reperfusion injuries were significantly less in females. Similarly, the protective effects of IPC were more pronounced in females. Our results showed that, with no major difference between male and female rats, IPC procedure reduces both BUN and creatinine levels in both male and female rats. Since, measurable changes in levels of BUN and creatinine occurs after severe damages to kidney tissue, thus, the results of the present study may be defensible and histological differences should be the base of judgment between two genders. 

## Conclusions

In this study, we concluded that the induction of IPC leads to improvement of kidney functions and also declines the injuries in both genders. However, female rats had better histological results that confirmed sex differences. According to our results and considering the findings of other studies, it is strongly recommended to apply the IPC process right before the transplantation and also choose females as donors. Therefore, post transplantation results would probably be more efficient and larger number of patients will survive. 
